# Taxonomic composition, community structure and molecular novelty of microeukaryotes in a temperate oligomesotrophic lake as revealed by metabarcoding

**DOI:** 10.1038/s41598-023-30228-4

**Published:** 2023-02-22

**Authors:** Konstantina Mitsi, Daniel J. Richter, Alicia S. Arroyo, David López-Escardó, Meritxell Antó, Antonio Guillén Oterino, Iñaki Ruiz-Trillo

**Affiliations:** 1 Institut de Biologia Evolutiva (CSIC-Universitat Pompeu Fabra), Passeig Marítim de La Barceloneta, 37-49, 08033 Barcelona, Spain; 2 Institut de Ciències del Mar (CSIC), Passeig Marítim de La Barceloneta, 37-49, 08033 Barcelona, Spain; 3 I.E.S. “Escultor Daniel”, C/ Gonzalo de Berceo, 49, 26005 Logroño, La Rioja Spain; 4 Institució Catalana de Recerca I Estudis Avançats (ICREA), Passeig Lluís Companys, 23, 08010 Barcelona, Spain

**Keywords:** Ecology, Microbiology, Limnology

## Abstract

Microbial eukaryotes are diverse and ecologically important organisms, yet sampling constraints have hindered the understanding of their distribution and diversity in freshwater ecosystems. Metabarcoding has provided a powerful complement to traditional limnological studies, revealing an unprecedented diversity of protists in freshwater environments. Here, we aim to expand our knowledge of the ecology and diversity of protists in lacustrine ecosystems by targeting the V4 hypervariable region of the 18S rRNA gene in water column, sediment and biofilm samples collected from Sanabria Lake (Spain) and surrounding freshwater ecosystems. Sanabria is a temperate lake, which are relatively understudied by metabarcoding in comparison to alpine and polar lakes. The phylogenetic diversity of microbial eukaryotes detected in Sanabria spans all currently recognized eukaryotic supergroups, with Stramenopiles being the most abundant and diverse supergroup in all sampling sites. Parasitic microeukaryotes account for 21% of the total protist ASVs identified in our study and were dominated by Chytridiomycota, both in terms of richness and abundance, in all sampling sites. Sediments, biofilms and water column samples harbour distinct microbial communities. Phylogenetic placement of poorly assigned and abundant ASVs indicates molecular novelty inside Rhodophyta, Bigyra, early-branching Nucletmycea and Apusomonadida. In addition, we report the first freshwater incidence of the previously exclusively marine genera *Abeoforma* and *Sphaeroforma.* Our results contribute to a deeper understanding of microeukaryotic communities in freshwater ecosystems, and provide the first molecular reference for future biomonitoring surveys in Sanabria Lake.

## Introduction

The tree of eukaryotes is an ideal playground for biodiversity explorers. Although land plants, animals and fungi initially attracted most of researchers’ attention, the advent of molecular techniques in biodiversity assessment has revealed an enormous diversity of microbial eukaryotes outside these three groups^1,2^. The paraphyletic assemblage of microbial eukaryotes is collectively referred to as protists^3^. Protists are valuable from an evolutionary perspective because by studying their life traits we gain insights into the evolutionary processes that shaped the extant eukaryotic tree of life^4–8^. In addition, protists are abundant, diverse and widespread organisms with key roles in important ecosystemic functions^9–11^. However, despite their importance in different ecosystems as producers^12^, grazers^13,14^, predators^15^ and parasites^16^, they have attracted less attention in comparison to their prokaryotic counterparts in environmental surveys^10^.

Sampling based on morphological identification combined with environmental DNA (eDNA) analyses^17^ have shown that protists are everywhere^18^. However, they are not everywhere equally studied. Microbial eukaryotes have been widely explored in marine ecosystems^19–27^, whereas there are fewer studies available regarding their distribution and diversity in soils^16,28–30^ and in freshwater systems^31,32^. Freshwater ecosystems are more fragmented and isolated^33,34^ in comparison to the open ocean where microbial communities are transported by currents on a global scale^35,36^. This intrinsic lower connectivity of freshwater ecosystems hinders the dispersal of freshwater organisms and increases the genetic diversity^37^.

Among freshwater habitats, lakes are undoubtedly the environments with the greatest number of molecular studies available^38–40^. High mountain lakes^40–43^ and polar lakes^44,45^ have been extensively studied due to their extreme conditions of temperature, nutrient availability and UV radiation. These systems have been shown to harbour a high proportion of unclassified sequences within numerous eukaryotic lineages. Fewer molecular biodiversity surveys, however, have been conducted in lakes of temperate areas^46,47^.

In this study, we explore the diversity of microbial eukaryotes in Sanabria Lake (Spain), a temperate lake at an altitude of 1004.1 m above sea level. Sanabria is an oligotrophic to oligomesotrophic, warm, monomictic lake with winter circulation and summer stratification. In comparison to lakes of other trophic states, oligomesotrophic lakes harbour the richest and most diverse lentic organismal communities^46^. Sanabria Lake is the biggest natural glacial lake in the Iberian Peninsula^48^ and has already been the subject of many traditional limnological studies^49–56^. However, no molecular data are currently available for this freshwater system.

The aim of this study is to explore the eukaryotic microbial community of Sanabria Lake using a massively parallel sequencing approach. To do so, we targeted the V4 hypervariable region of the 18S rDNA gene. We explored the taxonomic composition of the microbial eukaryotes present in the lake and the surrounding freshwater systems, including an exploration of the protist parasite diversity. We also assessed the community structure and the compositional heterogeneity across different sampling sites, habitats, and filter size fractions. Finally, we analysed our samples using a phylogenetic placement approach to quantify molecular novelty and we identified the branches of the eukaryotic tree that harbour potentially novel abundant taxa. Sanabria Lake is a protected biotope and it is under continuous monitoring. This is the first biodiversity study of Sanabria Lake that is based on molecular data, which will constitute a reference for future monitoring efforts.

## Materials and methods

### Study area

Sanabria Lake is situated in the NW of Spain (42 7′30″ N, 06 3′00″ W) between the provinces of León and Zamora at an altitude of 1004.1 m above sea level. It was formed by glacial erosion after the Würm glaciation in the Pleistocene, and it is the only lake formed by a terminal moraine in the Iberian Peninsula^57^. Sanabria Lake belongs to the Duero River Basin that has a total drainage surface of 127.3 km^2^^58^ and its main tributary is the Tera River. The surface of the lake is 3.46 km^2^^58^. It is divided longitudinally into two basins, one in the west with maximum depth of 46 m and another in the east with maximum depth of 51 m^48^. The shoreline length is 9518 m and the maximum width is observed in the eastern basin (1530 m)^48^. Regarding its mixing characteristics, Sanabria Lake is a warm, monomictic, holomictic lake^56^. The mixing period extends from late November to early March, when a thermocline normally appears^56^. No anoxic conditions have been observed in any layer of the water column during the thermal stratification^54,56,58^. Sanabria Lake is considered as oligotrophic to oligomesotrophic in view of its low levels of chlorophyll *a*, nutrient concentration, phytoplanktonic biovolume values and production rates^50,54–56,58^. The oligotrophic state of the lake is a result of its geology. Its drainage basin runs over an acid rock substrate (gneiss and granodiorites) of low solubility, making the water very poor in salts^59^. The lake is part of the Sanabria Lake Natural Park (BOE 1978), a protected area that supports a population of 2 small villages (~ 200 residents), one in the north and the other in the west side of the lake. During the summer, the National Park receives a high influx of tourists and there are three camping sites, all located on the east side of the lake. Since 2012, the Duero International Biological station has raised concerns that Sanabria Lake is undergoing an eutrophication process due to contamination from a deficient sewage depuration system^60^. However, studies based on pigment measurements and microscopy observation of the phytoplankton community do not support the eutrophication scenario and confirm the current oligotrophic state of the lake^54,55^.

### Sampling

Sampling was conducted at the beginning of the thermal stratification in March 2016. This time point was chosen because the physicochemical conditions of the lake are homogeneous after the winter mixing period, and it was expected that microbial eukaryotes would be homogeneously distributed across the lake, which would increase sampling efficiency. In addition, this time of year has the least anthropogenic impact, so any disturbance detected would indicate a permanent change rather than a temporal variation due to the presence of a stressor.

**Figure 1:**
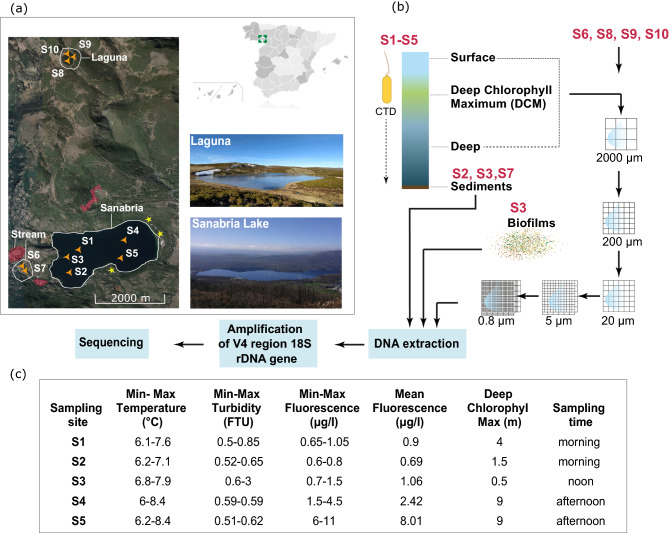
Sampling information. (**a**) The map on the top right shows the position of Sanabria Lake in the Iberian Peninsula. The satellite map (Source: Google Earth web, Imagery date: 12/07/2019, Camera: 16 km, Coordinates: 42°08′37″N 6°43′257″W) shows the study area. The sampling sites are pointed by orange triangles. Nearby villages are delimited by red coloured areas. Camping sites are pointed by yellow stars. (**b**) The sampling protocol. Filters of 2000 μm and 200 μm contained mainly multicellular organisms and were discarded. Sample S6 is water and S7 is sediment from an upstream tributary stream. Samples S8–S10 are water samples from a nearby small pond (Laguna de los Peces) that is not connected to the main water body. (**c**) CTD data collected in Sanabria Lake (sites S1–S5) during sampling.

To explore the diversity and the relative abundance of microbial eukaryotes in Sanabria Lake, 82 samples of water, sediment and biofilms from ten different locations were collected. Water samples were collected from five sampling sites in the lake basin (S1–S5), a tributary stream (S6–S7) and a nearby pond (S8–S10) (Fig. 1, Supplementary Table 1). A tributary is a stream or river that flows into a larger water body such as another river or a lake. A total of six different habitats were included in the sampling design: (i) two pelagic sampling sites in the lake, one in the west basin (S1) and another in the east basin (S4), (ii) the mouth of the River Tera (S2), which experiences the greatest anthropogenic impact in the studied ecosystem, (iii) a coastal area in the lake near two islets (Islas Moras) (S3), (iv) sulphurous waters in the east basin (S5), (v) water from a tributary stream (S6–S7) and (vi) water from three nearshore sites in a nearby pond (S8–S10). Water samples were collected from each of these habitats and were taken from the surface, the Deep Chlorophyll Maximum (DCM) and the deepest point above the sediment. The standard variables (turbidity, temperature, fluorescence) in the lake's main water body (S1–S5) were measured using a CTD SD204 (SAIV A/S) device. The water samples were prefiltered using filters of 2000 µm and 200 µm to remove debris and large multicellular organisms, and the size fractions above 200 µm were discarded and not included in the study. The water was then filtered sequentially using filters of 20 µm, 5 µm and 0.8 µm targeting the microplankton (20–200 μm), the nanoplankton (5–20 μm) and the pico/nanoplankton (0.8–5 μm) communities, respectively. In addition to water samples, sediments were collected from three sampling sites (S2, S3, S7) and 12 epilithic biofilm samples were collected from one sampling site (S3). All samples were placed in 2 ml cryovials and stored at − 80 °C until DNA extraction.

### DNA extraction, PCR amplification and sequencing

The filters were chopped using sterile scissors, and the sediments and biofilms were homogenised before further processing. Whole genomic DNA was extracted using the standard protocol of the PowerSoil DNA Isolation Kit (MO BIO). The V4 hypervariable region of the 18S rRNA gene was amplified using the universal eukaryotic V4 primers TAReuk454FWD1 (5′-CCAGCA(G⁄C)C(C⁄T)GCGGTAATTCC-3′) and TAReukREV3 (5′-ACTTTCGTTCTTGAT(C⁄T)(A⁄G)A-3′)^25^. The amplicons were sequenced using the Illumina MiSeq platform at Centro Nacional de Análisis Genómico (CNAG, Barcelona, Spain). The sequences were demultiplexed and the barcodes were trimmed by the sequencing facility. The raw sequencing data were submitted to the European Nucleotide Archive (ENA) under the accession number PRJEB23911 (ERP105690).

### Amplicon Sequence Variants (ASVs) analysis

The raw reads were analysed following a clustering-free approach. The Illumina demultiplexed paired-end sequenced dataset was processed using the R package dada2^61^. The read quality profiles were visualised using the function plotQualityProfile. The quality of the forward and reverse reads started decreasing after the position 250 and 240, respectively. The function filterAndTrim was used to discard low quality sequences using standard filtering parameters (maxN = 0, maxEE = c(2,2), truncQ = 2, rm.phix = TRUE, compress = FALSE, multithread = TRUE) and to trim the forward reads in the position 250 and the reverse reads in the position 240. The error model was calculated from our data with the function learnErrors and the estimated error rates were visualised with the function plotErrors. The reads were dereplicated using the function derepFastq and sequence-variants from our dereplicated data were inferred using the function dada. The forward and the reverse reads were merged to obtain the full denoised sequences using the default 12 bases overlap region and the paired reads that did not exactly overlap were removed. The amplicon sequence variant table (ASV) table was constructed using makeSequenceTable, then chimeras were removed with removeBimeraDenovo and finally the number of reads that made it through each step of our analysis was inspected (Supplementary Table 2). Taxonomy was assigned with the function assignTaxonomy that uses the Ribosomal Database Project (RDP) classifier together with the Protist Ribosomal Reference database (PR2) (v. 4.12.0)^62^ formatted for DADA2 (https://github.com/pr2database/pr2database/releases). An ASVs table that contains a total of 31,225 ASVs, the ASV counts per sample, and their taxonomy was generated using Biostrings::DNAStringSet and Biostrings::writeXStringSet from the Biostrings (version 2.58.0) R package in Bioconductor..

### Statistical analyses

The taxonomy, abundance, and metadata were combined to generate a phyloseq object^63^ (Supplementary Table 3). Different phyloseq datasets were generated by subsetting the initial phyloseq object (Supplementary Table 3) using the commands phyloseq::subset_samples and phyloseq::subset_taxa. Rarefaction curves were plotted using the function phyloseq::rarecurve to explore whether all included samples had reached saturation. Samples that did not reach saturation were removed. The phyloseq^63^, psadd (https://rdrr.io/github/cpauvert/psadd/), and Fantaxtic (https://rdrr.io/github/gmteunisse/Fantaxtic/) R packages were used to plot the taxonomic composition of the datasets.

Each dataset was rarefied at the minimum sample depth (Supplementary Table 3) in order to simulate even numbers of reads per sample. Rarefaction enables clustering samples according to their biological origin and permits fair comparison of diversity metrics among the samples^64^. Alpha and beta diversity were calculated in the subsampled datasets. Nine different alpha diversity metrics (Observed species, Chao1, se.chao1, ACE, se.ACE, Shannon, Simpson, InvSimpson, Fisher) were calculated using the function phyloseq::estimate_richnessin in order to examine whether alpha diversity estimates vary depending on the metric used. The significance of the difference in species richness was tested with pairwise comparisons using the Wilcoxon rank sum test, controlling for family wise error rate with the Holm–Bonferroni method^65^. Evenness was calculated according to Pielou^66^ and plotted as violin plots in the ggplot2 R package^67^. Beta diversity was measured using the Bray–Curtis dissimilarity statistic after converting ASVs abundances to frequencies within samples. To test the effects of habitat, sampling depth, sampling site, chlorophyll maximum and thermocline across samples, permutational multivariate analyses of variance (PERMANOVA) based on Wisconsin-standardized Bray–Curtis dissimilarities (Supplementary Table 6) was performed using the adonis function of the vegan package. Patterns of beta diversity were assessed via non-metric multidimensional scaling ordination (NMDS) also on Bray–Curtis dissimilarities using the function phyloseq::ordinate and were plotted using the function phyloseq::plot_ordination. The significance of groups revealed by NMDS was tested with analysis of similarity (ANOSIM) tests with 999 permutations (Supplementary Table 7).

### Phylogenetic novelty analysis

A phylogenetic placement approach was used to explore the molecular novelty present in the dataset. When taxonomy assignment based on sequence similarity methods fail, the most reliable way to classify an unknown sequence is by phylogenetic inference. However, large datasets of short-read query sequences produced by Illumina NGS technology such as the one generated by this study can not be analysed using traditional likelihood-based phylogenetic inference methods due to high computational complexity and lack of phylogenetic signal that results in poor branch support and biases such as long-branch attraction. Phylogenetic placement was proposed as a way to overcome these limitations and to bring the inferential power offered by likelihood-based approaches to large, short-read data sets^70^.

Two reference trees were built, one to encompass all eukaryotic diversity and another that was specific to encompass the extant diversity of unicellular Holozoa. The alignments for the reference trees were built with MAFFT v7.309^68^ with automatically selected strategy according to data size and were trimmed with trimal v1.4.rev15^69^ using the automated1 algorithm. The reference trees were constructed in RAxML^71^ under the GTRGAMMA model. Node support was estimated by 100 rapid bootstrap replicates. The query sequences (QS) were aligned to the reference alignments with PaPaRa (version 2.5)^72^. Phylogenetic placements were produced using RAxML-EPA^72^ and visualised with iTOL^73^.

## Results and discussion

### Abiotic parameters indicate ecological disturbance in the east basin of Sanabria Lake

We collected data on temperature, water turbidity, and chlorophyll *a* in order to describe the physical conditions of the lake at the time of sampling. We assessed the mixing state of the lake by temperature. Our aim was to collect the samples at the beginning of the thermal stratification when the eukaryotic microbial community is expected to be homogeneously distributed in the water body following the winter mixing. Water temperature at the surface ranged from 7.1 °C to 8.4 °C and in the deepest sampling points ranged from 6 °C to 6.85 °C, with a mean range of 1.62 °C (Fig. 1, Supplementary Table 1). These temperature measurements agree with the previously recorded temperatures during the homeothermic state of the lake that range between 4 to 7 °C^56^ and confirm the mixing state of the lake.

We assessed the trophic state of Sanabria Lake based on water turbidity and chlorophyll *a* values. Water turbidity is measured in FTU (Formazin Turbidity Units) and is an indicator of the trophic state of a lake as it is related to the concentration, type, and size of the suspended particles in the water^74^. During our sampling, turbidity values in Sanabria Lake were extremely low in all the sampling sites and ranged from 0.5 to 0.85 FTU (Fig. 1). These values are comparable to those in ultra-oligotrophic alpine lakes^75^. Chlorophyll *a* is a reliable indicator to assess the trophic state of a lake with high values to correspond to more eutrophic ecosystems^76^. Chlorophyll *a* mean values in Sanabria Lake have increased in the last fifty years (Supplementary Table 5) but they have not exceeded the levels that characterise oligotrophic lacustrine ecosystems. Together, these measurements confirm the overall oligotrophic status of the lake at the time of sampling.

We observed that chlorophyll *a* values differed between east and west basin during our sampling (Fig. 1). In Sanabria’s west basin (samples S1-S3), the mean value of chlorophyll *a* was below the reference value (1.5 μg/L). The reference value defines the equilibrium ecological state of the lake and confirms the absence of ecological disturbances. However, the mean values of chlorophyll *a* in Sanabria’s east basin (samples S4–S5) exceeded the reference values indicating the presence of ecological disturbance (Fig. 1). Values of chlorophyll *a* above 4.2 μg/L are linked to a Good-Moderate ecological state and values above 7.1 μg/L are linked to a Moderate-Poor ecological state^77^. Our results showed that there was some ecological disturbance that altered the values of chlorophyll *a* in the east basin of Sanabria Lake at the time of sampling. The altered values of chlorophyll *a* in the east basin may be related to higher anthropogenic impact due to the presence of three camping sites on this side of the lake. Chlorophyll *a* values measured in Sanabria´s east basin in March 2017^55^ are lower than the ones presented in our study, implying that the stressor was temporal and that water quality has been restored.

### The V4 hypervariable region captures the microeukaryotic diversity of Sanabria Lake

To characterise the diversity of the eukaryotic community in Sanabria Lake, we sequenced the V4 hypervariable region of the 18S small subunit (SSU) rRNA gene. We chose to sequence the V4 over other hypervariable regions of the 18S rRNA gene because it provides taxonomic resolution close to that of the full-length gene^78^ and it is the most suitable hypervariable region to use for phylogenetic placement^16^. A total of 15,947,744 reads from 82 samples were filtered, dereplicated and merged resulting in 31,225 Amplicon Sequencing Variants (ASVs). The study of multicellular organisms was out of the scope of the present work and thus most multicellular organisms were discarded by using physical filters of 2000 µm and 200 µm. However, some environmental DNA (eDNA) that originates from cellular material shed by multicellular organisms into the lake was sequenced together with the community DNA of unicellular eukaryotes. For our subsequent analyses, we bioinformatically filtered out all ASVs that were assigned to animals (Division/Class = Metazoa), land plants (Division = Streptophyta) and typical terrestrial fungi (Class/Order = Ascomycota, Class/Order = Basidiomycota) (Supplementary Table 3, dataset D3). After the removal of multicellular taxa, 27,790 microeukaryotic (protist) ASVs remained. We evaluated the sampling depth and the representation of microbial eukaryotes in our samples using rarefaction curves (Supplementary Figure 1). The curves reached a plateau for all samples, indicating that most of the microbial richness present in Sanabria Lake and the surrounding freshwater systems was successfully captured by our study.

### Spatial biodiversity patterns

**Figure 2:**
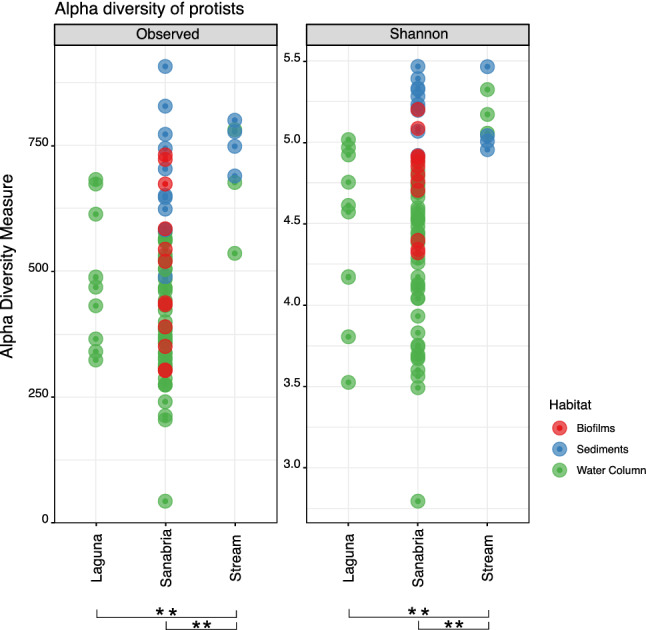
Alpha diversity of protists across the sampling sites. Each dot represents a sample and the colour code indicates the habitat of origin. Significant differences between pairs are indicated by double asterisks (p-value ≤ 0.01**).

To evaluate the intra-sample diversity of Sanabria Lake and the surrounding water bodies, we calculated nine different alpha-diversity indices (Supplementary Table 3). To avoid potential biases in diversity estimates due to differences in the total number of reads, we randomly subsampled the ASVs to the minimum depth of our dataset (Supplementary Table 3, dataset D3, min sample depth = 31,361 reads) before calculating the alpha-diversity indices. The number of total taxa reported was not affected by subsampling. We compared the diversity of the different water body types and we found that samples collected in the tributary stream showed significantly higher intra-sample diversity (Fig. 2) and greater evenness (Supplementary Figure 9) compared to samples from Laguna (pond) and Sanabria (lake) (Wilcoxon rank sum test P value < 0.01). Previous studies have shown that small water bodies like ponds and streams can contribute significantly to regional biodiversity of macrophytes and macroinvertebrates^79^. Our data support the hypothesis that the same is true for microeukaryotes. This result pinpoints the importance of small water bodies as biodiversity reservoirs and contrasts with their relative status in national monitoring and protection strategies, where they are frequently ignored. Regarding the different habitats, sediments harbour the richest microeukaryotic communities (Fig. 2). Sediments have been shown to harbour richer communities than the water column for other groups of organisms like bacteria^80^ and marine diatoms^81^. However, we cannot exclude that part of the diversity recorded in the sediments can be attributed to either dormant stages of planktonic microeukaryotes or dead cells that were recently settled from the water column.

To test the effect of abiotic factors in the protist community structure, we carried out permutational multivariate analysis of variance (PERMANOVA) using the Bray–Curtis dissimilarities of the ASVs between communities as a function of sample spatial origin (Supplementary Table 6). All factors tested by PERMANOVA tests revealed significant differences in protist communities as a function of site (Sanabria Lake, Laguna, Stream), sampling site (S1–S10), position regarding the chlorophyll maximum (on–off), position regarding the thermocline and habitat (water column, sediments, biofilms) (Supplementary Table 6).

**Figure 3:**
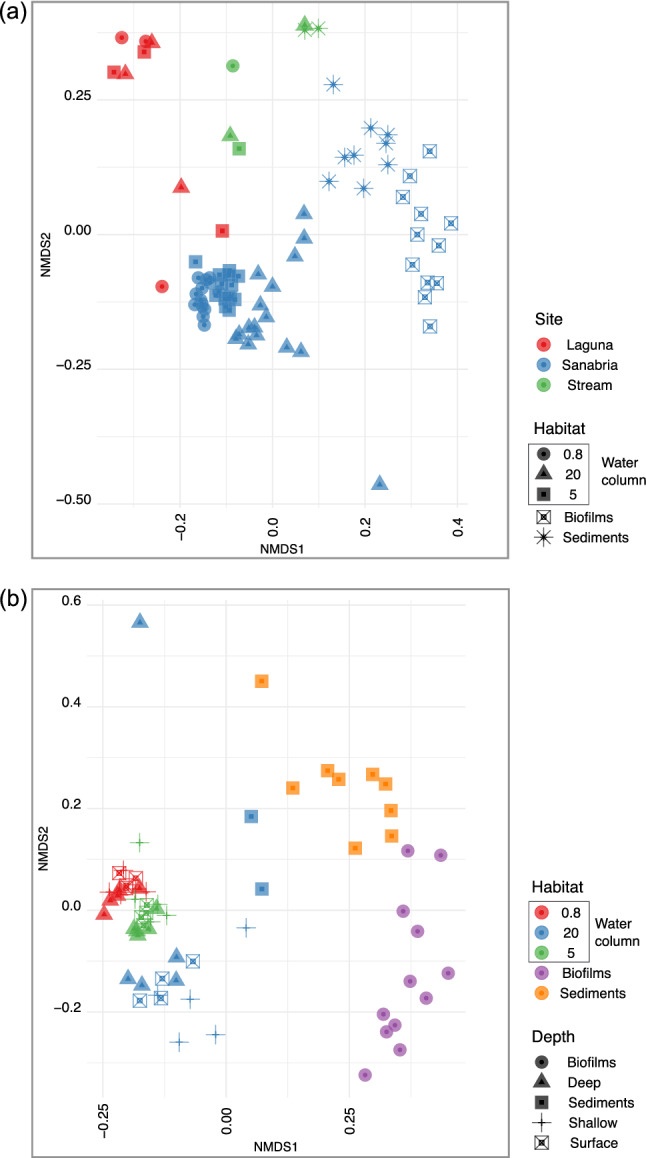
Beta diversity. Reduced-space NMDS plot showing microbial eukaryotes community structure based on Bray–Curtis dissimilarity. (**a**) Dissimilarity calculated from the rarefied at even depth (31,361 reads) abundances of protist ASVs in all samples (Supplementary Table 3, dataset D3). (Stress = 0.2083063, Procrustes: rmse 0.03612112 max resid 0.1863833), (**b**) Dissimilarity calculated from the abundances of rarefied at even depth (31,361 reads) ASVs present only in Sanabria samples (dataset D5) (Stress = 0.1676406, Procrustes: rmse 2.784844e−06 max resid 1.93149e−05).

To visualise the compositional differences in the community structure of protists we applied non-metric multidimensional scaling (NMDS). The communities from Sanabria Lake, the tributary stream and the Laguna were clearly separated in an ordination based on sampling site (Fig. 3a). In Sanabria Lake, the habitat was the main grouping factor of the microbial community structure, resulting in clustering of the communities from the water column, the biofilms, and the sediments (Fig. 3b). Furthermore, the community of microbial eukaryotes in the water column of Sanabria Lake was clearly segregated as a function of the filter size fraction and not the sampling depth (Fig. 3b). This is what we expected given that we sampled at the beginning of the thermal stratification after the winter mixing at the point of maximum homogeneity of the community. Our observations were statistically supported by ANOSIM tests (Supplementary Table 7), which showed significant and marked differences among communities according to habitat, sampling site, and depth (Supplementary Table 1).

Given that chlorophyll *a* values differed between east (S1, S2, and S3 sampling sites) and west basin (S4 and S5 sampling sites) (Fig. 1), we investigated whether this difference is reflected to the structure of their microeukaryotic communities (Supplementary Figure 2). For the purpose of this analysis we only included samples from the water column, because we do not have sediments and biofilms samples from the east basin and we already know that biofilm, sediments, and water column microeukaryotic communities significantly differ between them. Permutational multivariate analysis of variance showed that east and west basin water column microeukaryotic communities do not differ significantly between them (SumsOfSqs = 0.3077, MeanSqs = 0.30768, F.Model = 0.85978, R^2 = 0.02104, Pr(> F) = 0.619).

To conclude, our results suggest that the community structure in Sanabria Lake and the surrounding freshwaters is characterised by spatial variation. The habitat is a major factor that shapes the community structure after the winter mixing period. Sediments, biofilms, and water column harbour compositionally heterogeneous microbial communities that are driven by the unique environmental parameters that characterise them.

### Taxonomic composition of the protist community

**Figure 4:**
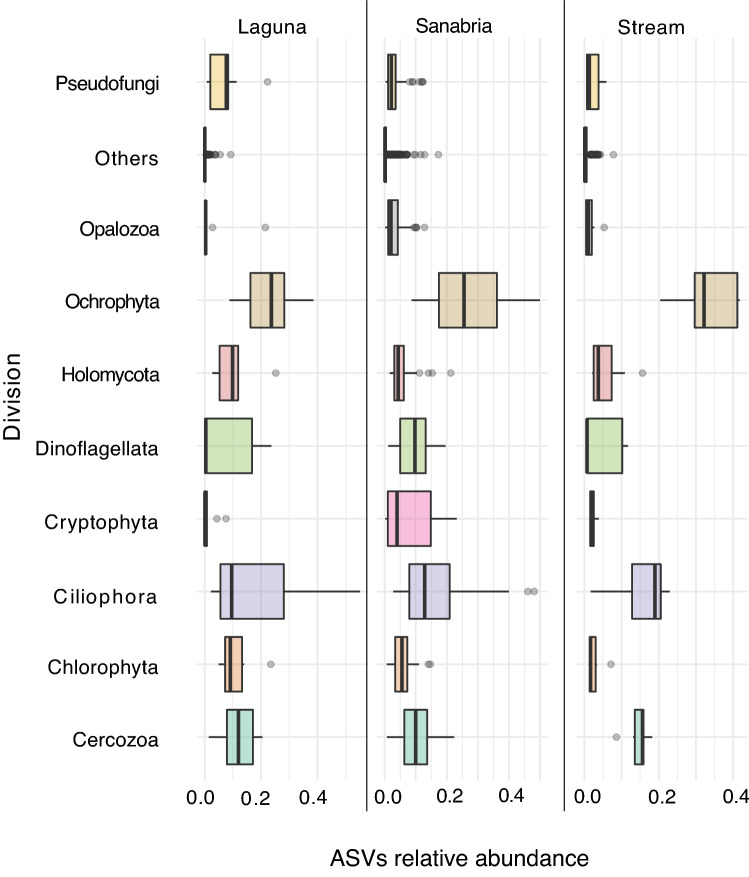
Distribution and relative abundance of the eukaryotic divisions across sampling sites. The taxonomy follows the system for the classification of protists proposed by Adl et al. in 2012 and implemented in the PR2 database by Guillou et al. in 2013. “Others” group together all taxa with relative abundance less than 1%. The boxes represent the interquartile range (IQR) between the first and third quartiles and the vertical line inside the box defines the median.

To gain an overview of the microeukaryotic taxonomic composition in the Sanabria Lake and the surrounding freshwater systems, we plotted the relative abundance of ASVs at division level (based on the PR2 taxonomy) across sampling sites (Fig. 4). The phylogenetic diversity of ASVs covered all currently recognized eukaryotic supergroups^82,83^. The group of Stramenopiles was the most abundant supergroup in all sampling sites, accounting for the 33% of total reads in Sanabria Lake, 34% in the nearby pond (Laguna) and 40% in the tributary stream respectively (Supplementary Figures 3, 4, and 5). In addition to being abundant, Stramenopiles were diverse, encompassing 22% of total ASV richness (6988 ASVs) (Supplementary Table 3). Among Stramenopiles, Ochrophyta was the most abundant group in all sampling sites (Supplementary Figure 6). Most Ochrophyta in the tributary stream (85%) and Laguna (81%) were affiliated with Chrysophyceae (Supplementary Figure 6), a group that is generally common in low-nutrient lakes^84^. In Sanabria Lake, together with the Chrysophyceae (36%), we report a high relative abundance of Bacillariophyta (37%) and Synurophyceae (24%) within Ochrophyta, two phototrophic lineages that produce silica skeletons or scales (Supplementary Figure 6). Alveolata was the second most abundant and diverse supergroup, accounting for 26–28% of the total eukaryotic reads in each site (Supplementary Figures 3, 4, and 5) and a total of 4609 ASVs in the study (Supplementary Table 3).

The plankton community of Sanabria Lake (excluding the surrounding freshwater systems) was dominated by Ochrophyta (in the Stramenopiles supergroup; 26%), Ciliophora (Alveolata; 14%), Dinoflagellata (Alveolata; 10%), Cercozoa (Rhizaria; 10%), Cryptophyta (10%), and unicellular Opisthokonta (7%) (Supplementary Figure 3). The presence of all these groups except for unicellular Opisthokonta was previously documented by light microscopy in Sanabria Lake^56^. We further explored the taxonomic composition of Sanabria Lake by separately examining benthic and pelagic samples. The taxonomic composition of the benthic protist community, as represented by ASVs in the sediments, was dominated by Stramenopiles (36%), Alveolata (29%), Rhizaria (13%), Opisthokonta (7%), Amoebozoa (5%), Archaeplastida (3%), Hacrobia (3%), and Excavata (3%). In contrast, the planktonic microbial community was characterised by the prevalence of Hacrobia (16%) as the third most abundant eukaryotic supergroup after Stramenopiles (31%) and Alveolata (28%). The planktonic Hacrobia^85^ in Sanabria Lake included Cryptophyceae (84%), Katablepharidophyta (13%), Centroheliozoa (1.5%), Telonemia (1%), and Haptophyta (0.5%). Excluding Cryptophyceae, this is the first record for these taxonomic groups in Sanabria Lake. (Katablepharidophyta were previously classified inside Cryptophyceae until electron microscopy and molecular phylogenies provided evidence to consider them as a separate taxonomic group^86^).

### Protist parasites in a temperate oligomesotrophic lake

Here we provide the first description of the taxonomic composition of the unicellular eukaryotic parasites (Supplememtary Table 3, dataset D6) present in Sanabria Lake, the biggest natural lake in the Iberian Peninsula. Parasitic unicellular eukaryotes modulate large scale ecological processes by regulating the abundance and dynamics of their host population^87^. As their study by microscopy is tedious, little was known about their prevalence in freshwater systems until the advent of metabarcoding^88^.

The parasites accounted for 21.3% (5925) of the total protist ASVs identified in our study. The parasitic community composition was dominated in all sampling sites by Chytridiomycota, whose relative abundance within parasitic taxa was 29% in the tributary stream, 32% in Sanabria Lake and 42% in Laguna. The prevalence of Chytridiomycota in the pelagic zone of lakes has been previously reported^89,90^. Chytridiomycota, which includes more than 1000 described species^91,92^, was also the most diverse group of parasites in our study, including more than 2200 of the 5925 total parasite ASVs, distributed among more than 50 genera . Almost half of the chytrids in terms of abundance identified in our study belonged to the order Rhizophydiales, that are host‐specific chytrids that infect various phytoplankton species, mostly diatoms^93,94^. The prevalence of Rhizophydiales in the Sanabria Lake ecosystem was not surprising given that they are the most common planktonic chytrids in lacustrine ecosystems^95^. A species of *Rhizophydiales* was probably the causative agent of a chytrid infection in Sanabria Lake in 2014 that diminished the population of the diatom *Tabellaria fenestrata* and controlled an algal bloom caused by eutrophication^55^. The relative abundance of Perkinsea, a group of parasitic alveolates, ranged from 13 to 18% of total parasite abundance across the different sampling sites. Perkinsea were previously considered as strictly marine parasites^96–100^ until molecular environmental surveys revealed an unprecedented diversity of these organisms in freshwaters^87,101–103^.

The parasitic community of each sampling site carried a unique taxonomic signature. The parasitic community of the tributary stream was characterised by a higher proportion of Apicomplexa (17%) and Labyrinthulomycetes (12%) in comparison to the other sampling sites. Most of the apicomplexan ASVs in the tributary stream fell into eugregarines, the most abundant apicomplexan group in environmental surveys^104^. Parasitic Stramenopiles (Pseudofungi), a significant component of freshwater ecosystems^105^, constituted the second most abundant group in Laguna and represented 20% of the Stramenopiles and 7% (76,070 reads) of all eukaryotes in this small pond (Supplementary Figure 6). Within the group of parasitic Stramenopiles (Supplementary Figure 6), there was observed a higher prevalence of Oomycetes that are common fish pathogens^106,107^ in Laguna in comparison to the other sampling sites. Finally, Sanabria Lake harboured a higher relative abundance of Ichthyosporea (12%, 96,491 reads) in comparison to the other sampling sites (Stream: 2%, Laguna: 1%). The majority of the Ichthyosporea in Sanabria were associated with the marine genera *Abeoforma* (69%), *Sphaeroforma* (17%) and *Pseudoperkinsus* (10%), none of them previously identified in a freshwater environment.

To confirm the identity of the ichthyosporean ASVs in Sanabria Lake we analysed them by phylogenetic placement. We compiled a dataset that encompassed all the extant diversity of unicellular Holozoa (n = 234). Half of the complete 18S rDNA gene sequences used to build the reference tree belonged to uncultured environmental taxa. A total of 132 ASVs identified as Ichthyosporea by the Ribosomal Database Project (RDP) classifier were placed into the 465 branches of the reference tree (Supplementary Figure 7). Most of the queries were placed in a clade formed by the freshwater anuran parasite *Anurofeca richardsi*, the marine *Creolimax fragrantissima*, *Pseudoperkinsus tapetis* and *Sphaeroforma arctica*, and some uncultured environmental taxa (Supplementary Figure 8). The 132 ichthyosporean queries were clustered into 17 clades in the best-hit placement tree (Supplementary Figure 8). Most of the clades were associated with freshwater sequences. Clade 4, the one formed by the larger number of sequences, was assigned to the FRESHIP2 group^108^, expanding the known molecular diversity of these environmental taxa. Clades 13, 14 and 15 were assigned to *Anurofeca richardsi* and clade 9 to *Caullerya mensii*, another freshwater parasite that infects *Daphnia pulex*^109^. We identified two clades that were directly associated with marine Ichthyosporea, clade 6 that branched as sister to *Abeoforma whisleri* and clade 16 that branched as sister to *Sphaeroforma arctica* (Supplementary Figure 8). The genera *Abeoforma* and *Sphaeroforma* were previously considered exclusively marine and this is the first record that connects these taxa to freshwater habitats. As freshwater habitats are increasingly explored by molecular means, the number of taxa that have been previously reported as exclusively marine and later were found in freshwater surveys continues to expand^87,110–114^.

### Abundant and potentially novel freshwater microbial eukaryotes

Metabarcoding biodiversity studies have shown that a great part of the extant microbial diversity remains undocumented^1,2^. In a metabarcoding survey, a species can be described as novel either because it is completely unknown to science or because the particular molecular marker database used in the study does not include available information on the species. In this study, we use the term ‘novelty’ to describe ASVs that are not present in our reference database as it is not possible to know for certain at this point whether or not these ASVs represent known but unsequenced species.

To check whether Sanabria Lake and its surrounding freshwater systems could be a potential sampling site to isolate new organisms, we investigated the molecular novelty by first selecting potentially novel ASVs. We used the Ribosomal Database Project (RDP) classifier^115^ to assign taxonomy to the ASVs. The RDP classifier provides for each ASV an assignment of the best matching taxa together with a bootstrap confidence score at each taxonomic rank. This score represents the level of confidence of the taxonomic assignment at each rank, from supergroup to species. Here, we define as poorly assigned, thus potentially novel, all ASVs with bootstrap confidence score value < 97 at the supergroup level. We were interested in identifying the most abundant and novel microbial eukaryotes in our study site, so we selected all ASVs with more than 1000 reads and bootstrap confidence score value lower than the aforementioned established novelty threshold.

**Figure 5:**
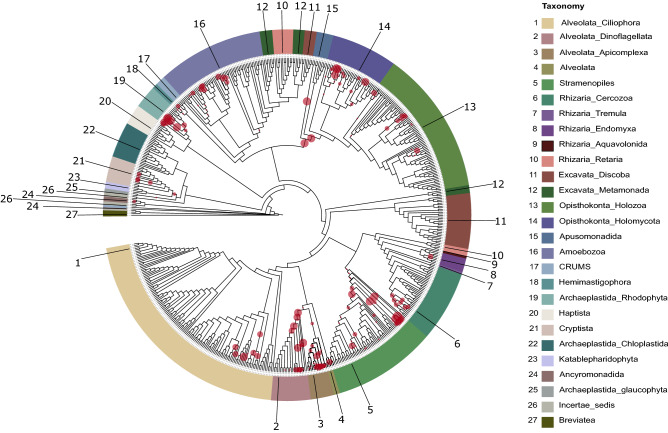
Novelty estimation. The tree shows the result of the phylogenetic placement of 113 ASVs into 1233 branches of a reference tree with 618 taxa. The reference taxon sampling spans all extant eukaryotic diversity as described in Adl et al. 2019. The diameter of the circles indicates the number of ASVs placed in each branch. The same ASV can be placed on multiple branches until its accumulated likelihood weight ratio reaches a value close to 1. Taxonomic groups with high number of placements in interior nodes indicate potential novel undescribed molecular diversity.

To assign taxonomy to the queries of our dataset, we analysed them using phylogenetic placement (Fig. 5). We first constructed a comprehensive eukaryotic reference tree with 618 eukaryotic taxa that encompassed all the extant eukaryotic diversity according to the latest classification of eukaryotes^83^. We designed the reference tree with two criteria. First, to be inclusive in order to minimise phylogenetic placement artefacts related to taxonomic sampling and second to be non-redundant in order to be smaller and thus easier to handle in the post placement analyses. The amplicon short sequences were aligned to the reference alignment and the amplicon sequences that were not aligned in the V4 region were removed as artefacts after manual inspection. We placed a total of 113 ASV V4 queries into 1233 branches of the reference tree.

Most of the ASV placements in the tree were found in the leaf nodes of Rhodophyta (Archaeplastida), Bigyra (Stramenopiles), early-branching Nucletmycea (also known as Holomycota), and Apicomplexa (Alveolata), pinpointing these clades as parts of the tree with potential novel undescribed molecular diversity (Fig. 5). An elevated number of placements was spotted in the internal nodes of Dinophyta and the divergence between Opisthokonta and Apusomonadida (Fig. 5). Apusomonadida is a recently defined phylum with a key phylogenetic position to understand the origin of the eukaryotic cell. Apusomonads are rarely detected in environmental studies^116–120^ and can be considerably more diverse than is currently perceived^121^. We report previously undocumented diversity associated with the genera *Cryptomonas* and *Chilomonas* inside Cryptista, the naked filose amoebae of the genus *Vampyrella* (Endomyxa), and the frequently detected by 18S rRNA gene sequencing eukaryovorous biflagellate Aquavolon^122^. No placement was recorded inside the group of Excavata.

## Conclusions

Metabarcoding analyses of the V4 hypervariable region of the 18S rRNA gene from diverse habitats in Sanabria Lake and the surrounding freshwater ecosystems uncovered a rich and diverse microeukaryotic community. One fifth of the diversity of microeukaryotes identified in Sanabria Lake are parasites, stressing the importance of parasitic taxa in the freshwater ecosystems. Our observations regarding the taxonomic composition of the microeukaryotic community overlap with microscopical data based on morphology but expand the total biodiversity recorded in the lake by adding taxa that were either insufficiently abundant to be detected by traditional methods or inconspicuous due to lack of taxonomically informative morphological characters. Tributary stream samples were significantly more species-rich than samples from Sanabria lake and Laguna. We found that sediments harbour the greatest diversity among different habitats. We observed compositional heterogeneity among the microbial communities of Sanabria Lake and the surrounding freshwater ecosystem. Phylogenetic placement analysis showed that Sanabria Lake and the surrounding freshwater ecosystems would be good targets for future studies aiming the discovery of potential novel microeukaryotes. This is the first metabarcoding record of the diversity in Sanabria Lake. Our results expand our understanding of the microbial communities in oligomesotrophic, temperate, lacustrine ecosystems and can be used as reference for future studies in the area.

## Supplementary Information

Supplementary Information.

## Data Availability

Raw data are available in the European Nucleotide Archive (ENA) under the accession number PRJEB23911 (https://www.ebi.ac.uk/ena/browser/view/PRJEB23911). Supplementary materials are available as figshare public repository under the https://doi.org/10.6084/m9.figshare.21884934. The code is available at https://github.com/kcmitsi/microeuk_Sanabria.
